# Scanning Acoustic Microscopy and Time-Resolved Fluorescence Spectroscopy for Characterization of Atherosclerotic Plaques

**DOI:** 10.1038/s41598-018-32788-2

**Published:** 2018-09-26

**Authors:** Bukem Bilen, Belkis Gokbulut, Ulku Kafa, Emre Heves, Mehmet Naci Inci, Mehmet Burcin Unlu

**Affiliations:** 1 Bogazici University, Physics Department, Istanbul, 34342 Turkey; 2 Mehmet Akif Ersoy Thoracic and Cardiovascular Surgery Training Research Hospital, Istanbul, 34303 Turkey; 3 Quantag Nanotechnologies, Istanbul, 34746 Turkey; 4 Global Station for Quantum Medical Science and Engineering, Global Institution for Collaborative Research and Education (GI-CoRE), Hokkaido University, Sapporo, 060-8648 Japan; 5 Department of Radiation Oncology, Stanford University School of Medicine, Stanford, CA USA

## Abstract

Atherosclerotic plaques constitute the primary cause of heart attack and stroke. However, we still lack a clear identification of the plaques. Here, we evaluate the feasibility of scanning acoustic microscopy (SAM) and time-resolved fluorescence spectroscopy (TRFS) in atherosclerotic plaque characterization. We perform dual-modality microscopic imaging of the human carotid atherosclerotic plaques. We first show that the acoustic impedance values are statistically higher in calcified regions compared with the collagen-rich areas. We then use CdTe/CdS quantum dots for imaging the atherosclerotic plaques by TRFS and show that fluorescence lifetime values of the quantum dots in collagen-rich areas are notably different from the ones in calcified areas. In summary, both modalities are successful in differentiating the calcified regions from the collagen-rich areas within the plaques indicating that these techniques are confirmatory and may be combined to characterize atherosclerotic plaques in the future.

## Introduction

The identification of atherosclerotic plaques as vulnerable is still under investigation since the roles of markers of plaque vulnerability are not entirely understood and defined. Thin-cap fibroatheromas (TCFAs), which are fibroatheromas with a fibrous cap thickness of <65 *μ*m^1^, are found to be at high risk for rupture. TCFA has a necrotic core and also calcium deposition^2,3^. Calcification is considered to be an active process which involves information about vulnerability^4^. In fact, microcalcification or spotty calcification within the plaque is found to be the precursor of cardiovascular complications^5^. Plaques with millimeter-sized or larger calcifications are said to be no longer inflammatory and stable^6,7^. Conventional noninvasive imaging methods, such as computed tomography (CT) and echocardiography, can detect advanced calcifications. Meanwhile, magnetic resonance imaging (MRI), micro-optical coherence tomography (micro-OCT) or positron emission tomography (PET) can identify early calcifications despite certain limitations^6,8^. These modalities are either very expensive or involve ionizing radiation. No single modality with good enough penetration depth can give both morphological and chemical information about tissues at subcellular level^9^. Therefore, multimodal imaging, which combines two or more modalities with a reasonable cost, or a new intravascular technique can be a solution. Ultrasound imaging has become very popular for the observation of soft tissue since it has high axial and lateral resolutions of around 20–100 *μ*m, a good penetration depth of around 5 mm, and low cost, but it can only provide morphological information. Besides, for the detection of microcalcifications, the signal detection capability of conventional ultrasonography has to be increased, since high echo signals from such small surfaces are not available^10^. These disabilities have been overcome by combining ultrasound with photoacoustic imaging and the detection of lipid-laden plaque was achieved by providing both morphological and lipid-specific chemical information about the human coronary artery^11^.

Scanning acoustic microscopy (SAM) is another imaging tool which gives information about the morphology and the mechanical properties of the specimen simultaneously at microscopic levels. Focused high-frequency ultrasound is used to identify the elastic properties of biological tissues. SAM has two major advantages over other imaging techniques; it does not require special staining, and it obtains the image of an area of around 5 mm × 5 mm in a couple of minutes. SAM calculates either the speed of sound (SOS) through tissues^12–21^ or acoustic impedance of samples^22,23^ and two-dimensional distributions are mapped. Similarly, acoustic microscopy can resolve cells and organelles using higher frequencies of 100 to 1200 MHz^24–31^.

Time-resolved fluorescence spectroscopy (TRFS) measures the average fluorescence lifetime a fluorophore in a biological tissue spends in the excited state when it is excited by a source from its ground state. The lifetime of the fluorophore changes as a result of interaction with the molecular environment. A change in the lifetime allows us to analyze various parameters regarding the molecular environment such as binding, temperature or concentration. Even though TRFS has not been implemented in clinics yet, it has been investigated widely as a new tool for the characterization of atherosclerotic plaques. Its success in obtaining compositional information about the plaques^32–34^ inspired scientists to combine fluorescence lifetime imaging with other modalities such as IVUS^35^, second harmonic generation (SHG) microscopy^36^, OCT^37^ and Raman spectroscopy^38^. Intravascular catheter studies using TRFS technique have also been done^39,40^, since, TRFS catheters can obtain a good signal even within the artery, where, blood does not affect lifetime properties but fluorescence intensity.

Here, we perform SAM and TRFS measurements to characterize the atherosclerotic plaques. We test the ability of these modalities in resolving the collagen-rich areas from calcified regions. While SAM provides micrometer resolution structural and mechanical information regarding the plaques, TRFS provides information about the molecular environment of the plaque. Our study shows that acoustic impedance microscopy is capable of identifying calcifications within the human carotid atherosclerotic plaques, which are signs of vulnerability at the microscopic level. TRFS results confirm these results of SAM by the measurement of noticeably different decay parameters at collagen-rich and calcified regions. Therefore, we conclude that the combination of SAM with TRFS in the future will enable these imaging techniques to be used simultaneously for the determination of plaque vulnerability in clinics.

## Results

### Acoustic Impedance Microscopy Results

**Figure 1:**
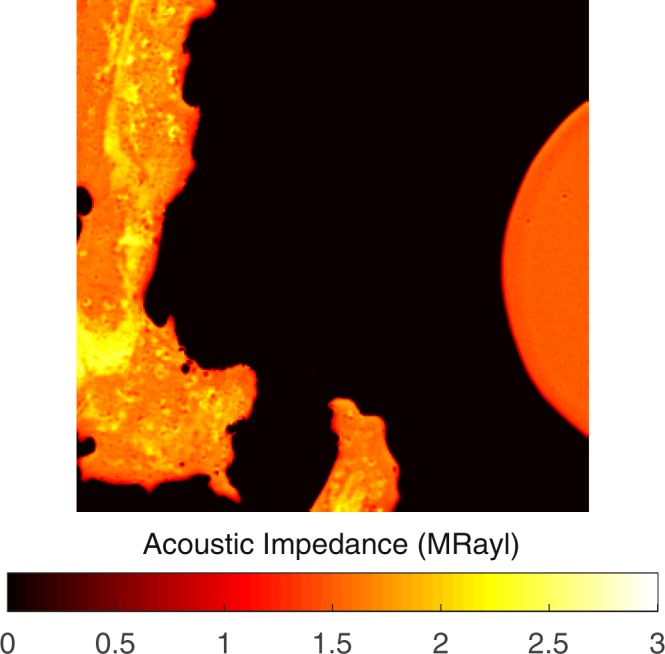
Acoustic impedance map of an atherosclerotic plaque obtained by comparing the reflected ultrasound signals from the surfaces of water and the sample. The scanning area is 4.8 mm × 4.8 mm.

The plaques received from patients undergoing carotid endarterectomy operation had cylinder-like structures of a height of around 2 cm and radius of approximately 1 cm. By slicing them with a lancet, we prepared smaller cylindrical cross sections of a height of 2 mm and radius of 1 cm with plain facets, for SAM studies. All of the carotid atherosclerotic plaques were received from patients older than 50 years of age and therefore, had advanced lesion type (fibrocalcific). We obtained the images using acoustic impedance mode of SAM. Figure 1 shows the acoustic impedance map of one sample. This image was constructed using the acoustic reflections from both surfaces of the reference (water) and the plaque cross-section on the polystyrene substrate. The acoustic impedance distribution indicated different acoustic properties due to the variation of elasticity within the atherosclerotic plaques. The acoustic impedance was determined to be less than 2 MRayl for the collagen-rich areas and greater than 2 MRayl for the calcified areas. In the intensity image, shown in Fig. 1, the reflected signals from calcified regions were brighter than the ones from collagen-rich regions since the brightness of the intensity images directly depend on the ultrasound intensity reflected, which was greater from a more rigid surface than from a softer surface.

**Figure 2:**
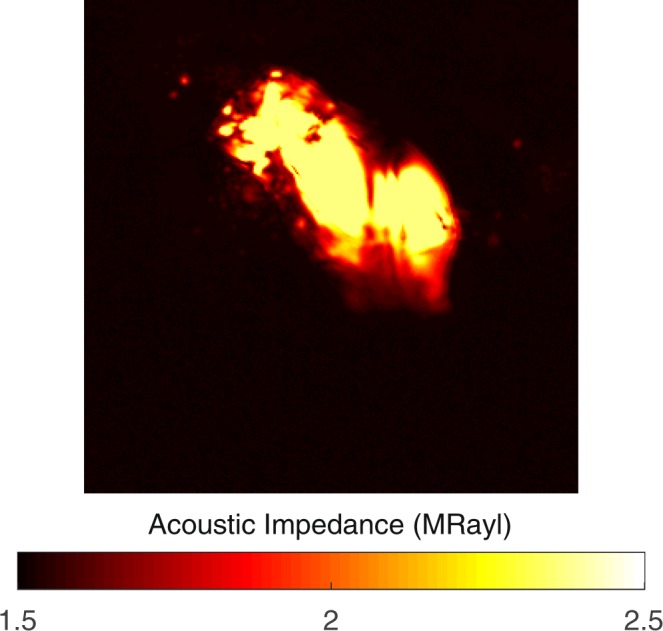
Acoustic impedance map of a fibrocalcific plaque obtained with the alternative sample setup, in which the acoustic waves are reflected directly from the sample’s surface without passing through the substrate. The scanning area is 4.8 mm × 4.8 mm.

The measurements were repeated with an alternative sample setup in which the plaque samples on polystyrene substrates were turned upside down and acoustic waves were reflected back directly from sample’s surface without passing through polystyrene substrate. Acoustic impedance and intensity maps of the plaque sample were obtained successfully, also with this sample setup, as shown in Fig. 2. Fibrocalcific plaques mainly had two areas; collagen-rich and calcific with acoustic impedance values of less than 2 MRayl and higher than 2 MRayl, respectively.

**Figure 3:**
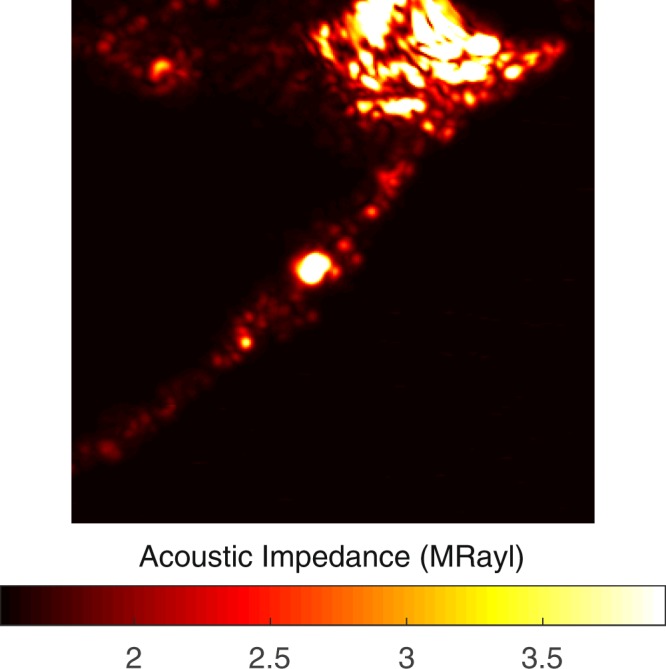
Acoustic impedance map of a severely calcific plaque sample obtained with the alternative sample setup. The scanning area is 4.8 mm × 4.8 mm.

Images of a severely calcific sample, in Fig. 3, were obtained with the alternative sample setup. The sample had a large calcification ratio and collagen content was not significant, therefore, the impedance value was greater than 2 MRayl almost everywhere within the sample.

Table 1 summarizes acoustic impedance microscopy results of all plaques studied in this investigation. The values obtained by both sample setups are identical. Calcific regions are dark-colored and therefore easily distinguishable. The fibrocalcific samples have also collagen-rich regions, not lipids, therefore the remaining area belongs to the collagen-rich region. Collagen-rich and calcific regions were distinguished easily with significantly different acoustic impedance values. Each value given in Table 1 is the average obtained from multiple points within each area.

**Table 1:** Acoustic impedance values of carotid atherosclerotic plaques of 10 different patients with two distinguished regions of calcification and collagen.

Patients (Age, Gender)	Acoustic Impedance (collagen-rich region) (MRayl)	Acoustic Impedance (calcific region) (MRayl)
1–63, Female	1.85 ± 0.08	2.08 ± 0.07
2–62, Female	1.87 ± 0.08	2.24 ± 0.10
3–79, Male	1.78 ± 0.17	2.39 ± 0.10
4–85, Male	1.80 ± 0.18	2.30 ± 0.10
5–75, Male	1.75 ± 0.24	2.51 ± 0.18
6–74, Male	2.10 ± 0.21	3.95 ± 0.01
7–62, Female	1.85 ± 0.09	2.12 ± 0.09
8–66, Female	1.83 ± 0.10	2.56 ± 0.13
9–59, Female	1.83 ± 0.18	2.16 ± 0.12
10–63, Male	1.78 ± 0.07	2.37 ± 0.01

5 female and 5 male patients with divergent ages were studied for the correlation of age and gender on plaque contents.

### TRFS Results

After SAM experiments were completed, plaque samples were painted with CdTe/CdS quantum dots for time-resolved fluorescence spectroscopy. The diluted aqueous solutions of CdTe/CdS quantum dots, after being stirred firmly to avoid precipitate and aggregate formation, were dropped with a dosimeter on each sample equally. The mean lifetime of the quantum dots on a microscope slide, calculated as 9.24 ± 0.01 ns, was measured for comparison with the decay lifetime values of dots on the plaque samples. In Table 2, the fluorescence decay parameters of the quantum dots on atherosclerotic plaques were given. Each value given in Table 2 is the average of all samples obtained from multiple points within each area. Around calcifications, average lifetime was the smallest, while it was higher around collagen-rich regions and in several samples reached the highest value at the outermost layers.

**Table 2:** Average lifetime values of CdTe/CdS quantum dots on calcific, collagen-rich and outermost regions of all atherosclerotic plaques.

Region	<*τ* > (*ns*)
Calcific	1.45 ± 0.27
Collagen-rich	2.54 ± 0.23
Outermost	3.59 ± 0.22

**Figure 4:**
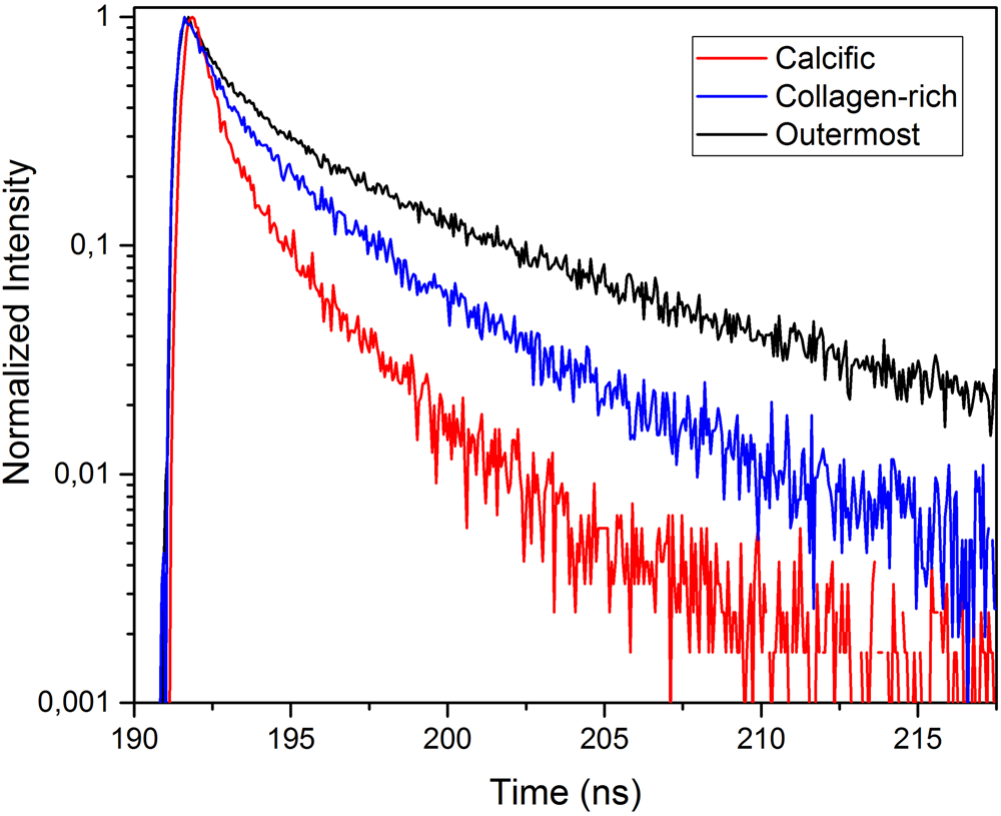
Fluorescence decay curves of CdTe/CdS quantum dots on calcific, collagen-rich and outermost regions of atherosclerotic plaques.

The fluorescent decay curves, in Fig. 4, were obtained by the excitation of the CdTe/CdS quantum dots on three regions of the plaque samples, whose decay parameters were given in Table 2. For these different regions of the sample, the decay parameters of the quantum dots were calculated by the two exponential decay fit, which minimized the *χ*^2^ parameter. A significant change was observed in the characteristics of the decay curves for various regions of the sample. These results clearly proved the existence of an obvious and efficient electron transfer between the quantum dots and the plaque.

## Discussion

We have shown that SAM can differentiate morphological and mechanical properties of collagen-rich and calcified regions of the atherosclerotic plaque samples by measuring the acoustic impedance values. The impedance values are higher in the calcified regions than in the collagen-rich regions. The high acoustic impedance is a result of accumulation of calcium. Calcific regions are dark-colored, in fact, in some samples they are black due to former harmless thrombosis [3], therefore easily distinguishable. The fibrocalcific samples have collagen-rich regions in addition to calcifications, not lipids, therefore the remaining area belongs to the collagen-rich region. The calcified regions of the atherosclerotic plaques are also stiff with high elastic moduli. Some plaque samples have more severe calcifications than the others (Sample 6), therefore, their acoustic impedance values are even greater in calcified regions. In addition to this, it is very hard to distinguish collagen-rich regions in such samples and as a result standard deviation is high. The difference in the plaque samples is because the patients had undergone surgeries after either they experienced a cerebrovascular incident or had 70% and above shrinkage in their internal carotid artery (ICA), without any further information about the history of the pathology of the plaques and their progression. Besides, some patients are asymptomatic and they have been diagnosed by chance. In some plaque samples, we observed dark colored regions, which were sites of healed plaque ruptures. In 60% of sudden deaths with acute plaque rupture, healed plaque ruptures have been observed^41^.

The success of SAM in determining the calcific regions is promising for the diagnosis of patients with potentially vulnerable plaques *in vivo*. Two dimensional maps, as in Fig. 1, obtained with micrometer resolution would be sufficient to determine microcalcifications, which are indications of vulnerability, long before the plaques become stable^5^. The SAM measurements done with the conventional sample setup were problematic for calcific regions, since these rigid regions did not adhere to the substrate surface easily. Therefore, we acquired SAM images after applying a small amount of force onto them. The alternative sample setup in SAM was used for the first time for the atherosclerotic plaques and was more successful in the determination of different components proven by smaller standard deviations, especially in calcific regions (Sample 6 and Sample 11). When compared with the conventional setup, brighter images were observed, since ultrasound signals were reflected directly from the surfaces of the samples without attenuation in the substrates. In fact, this study with this sample setup was preliminary for intravascular studies, since a catheter would detect the plaques *in vivo* inside a vessel with blood flow, and there, blood would be the coupling medium, whereas here, water was the coupling medium.

The fluorescence lifetime values in different regions on the plaque samples were changing due to collagen, lipid or calcification content^35,38,40^. Detection of lipid accumulation, before the formation of fibrous lesions with high collagen content, was crucial for early stage diagnosis of atherosclerosis^3^, but for the fibrocalcific plaque samples, mainly, calcification with high collagen content was observed and lipid pools were not significant. The observation of fibrocalcific samples by TRFS resulted in discrimination of these two dominant regions and was confirming the results of SAM experiments. CdTe/CdS quantum dots were used on plaque samples to differentiate these different regions, since the laser source in TRFS experiments was suitable to excite these dots. Mean fluorescence lifetime of the CdTe/CdS quantum dots on collagen-rich and calcified regions were measured as 2.54 ± 0.23 ns and 1.45 ± 0.27 ns, respectively. The fluorescence decay parameters of quantum dots on the outermost layers of a couple of samples were even higher than the ones in the collagen-rich regions, possibly due to existence of normal artery tissue with some lipid content. Mean fluorescence lifetime of quantum dots on outermost layers was measured as 3.59 ± 0.22 ns. It is known that some excitation wavelengths might cause fluorescence of intrinsic fluorophores. In our experiments, we were confident about our single photon counting system which monitored CdTe/CdS emission only, since as the emission of CdTe/CdS quantum dots was blocked, no signal was detected. Another observation with fibrocalcific plaques was that, as the calcification increased, lower lifetime values were measured, therefore, standard deviation in lifetime values around calcified regions was higher due to different calcification ratios of samples from different patients.

All of the carotid atherosclerotic plaques, received from patients undergoing carotid endarterectomy operation, were fibrocalcific with an insignificant lipid content, therefore, in this study, we were able to differentiate mainly collagen-rich and calcific regions by SAM and TRFS. Besides, we only studied plaque samples with no or very few normal tissue, therefore, we were unable to compare our results with measurements from a normal artery tissue. 5 male and 5 female patients with divergent ages were studied for the correlation of age and gender on fibrocalcific plaque contents. As can be seen in Table 1 the highest calcification, therefore the highest acoustic impedance, was not observed in the oldest patient (85 years old). Besides, there are no significant differences between acoustic impedance values of male and female patients’ specimens, indicating no sex dependence on the contents of the fibrocalcific plaques. We assume that these observations are due to the fact that atherosclerosis development depends on also other parameters like genetic tendency or lifestyles.

Even though CdTe/CdS quantum dots yield impressive results for different regions of the plaques, as can be seen in Fig. 4, they are toxic and cannot be used in clinical applications. Therefore, instead of CdTe/CdS quantum dots, graphene-based quantum dots, that are biocompatible in the human body, can be used^42^. Functionalized quantum dots were observed to be more stable and highly accumulated within the targeted component of plaques, so they have great potential in plaque-type detection.

In this article, we evaluate the consistency of the results of SAM and TRFS in the characterization of atherosclerotic plaques for the first time and discuss the abilities of these imaging modalities on the determination of plaque components. The characterization of the atherosclerotic plaques by SAM with a new sample setup is done also for the first time so far and the results are even more successful than the results of conventional setup. Therefore, in this study, this new sample setup has proven itself in the determination of atherosclerotic plaque components. The determination of collagen and calcification within the fibrocalcific plaques was done successfully by SAM and TRFS. Acoustic impedance maps of the samples show clearly different values in collagen-rich and calcified regions. Similarly, lifetime values of CdTe/CdS quantum dots in these regions differ noticeably. Consequently, the combination of SAM with TRFS has a potential to be used in clinics and will enable early diagnosis of vulnerable plaques *in vivo* with a verification, since both modalities are capable of acquiring morphological and chemical information about the plaques. However, for combining SAM with TRFS, first, an intravascular SAM probe, similar to intravascular ultrasound (IVUS) probe, has to be developed. Then, it can be combined with the fluorescence lifetime technique on a catheter system for *in vivo* studies. Ultrasound signals and excitation light will be sent onto the tissue under investigation. Reflected ultrasound signals and emission of quantum dots will be collected and then analysed for obtaining affirmative morphological and mechanical information.

### Study Limitations

Formalin fixation with 10% buffered formalin caused hardening in tissues when compared to fresh ones, due to cross-linking of proteins^12^ and therefore, exhibiting higher sound of speed values as the duration of fixation increases. In our study, all the specimens were fixed with a less concentrated formaldehyde of 4%, therefore, we assume less influence in our SAM and TRFS measurements.

We tried to inhibit fluorescence resonance energy transfer (FRET) between CdTe/CdS quantum dots by diluting its aqueous solution and comparing the TRFS results obtained for each concentration. A significant change was observed between highly concentrated and dilute solutions of CdTe/CdS, while, as the quantum dot solution further diluted, the difference in the results disappeared.

## Methods

### Specimens

Carotid atherosclerotic plaques were received from patients undergoing carotid endarterectomy operation. From each participant, informed consent was obtained for this study, which was ethically approved by Istanbul Mehmet Akif Ersoy Thoracic and Cardiovascular Surgery Training Research Hospital Ethics Committee (Number: 2017-18). The surgeries were performed under general anaesthesia. Longitudinal cervical incision was performed and exposure of the carotid bifurcation was obtained by division of the facial vein. Heparin 5000 U was administered intravenously (IV). Internal carotid artery (ICA), common carotid artery (CCA) and external carotid artery (ECA) were occluded with vascular clamps. An arteriotomy was made starting from CCA in the proximity of the lesion, extending through ICA, until a point where ICA was relatively normal. A temporary carotid balloon shunt was inserted. An elevator was used to take off the plague. The arteriotomy was closed with a Dacron patch. Routine closure of subcutaneous tissues and skin was performed. Plaque samples were fixed with 4% formaldehyde and were sliced approximately 2 mm thick cross sectionally for imaging by both modalities. Just before SAM and TRFS experiments, cross-sections were washed thoroughly with saline.

### CdTe/CdS Quantum Dots

A modified one-pot method was used in the synthesis of cadmium telluride (CdTe) quantum dots. First, sodium hydrogen telluride (NaHTe) was synthesized and during its synthesis, tellurium (Te) powder was reduced with sodium borohydrate (NaBH_4_). 0.0918 g Te powder and 0.06 g NaBH_4_ were inserted in a 50 ml reaction flask and purging with inert gas was started. After 1 h of purging, 20 ml of purged distilled water was poured into this reaction flask and heating started. At 50 °C the system was kept under inert atmosphere for 2 h to obtain a purple-colored solution. Then, inside a 250 ml two-necked flask, 3.12 mmol of cadmium chloride (CdCl_2_) and 420 *μ*l of thioglycolic acid (C_2_H_4_O_2_S) were dissolved with 110 ml of ultra-pure water. By dropwise addition of 1.0 M sodium hydroxide (NaOH) solution, pH of the solution was set to 11.0–11.5. For purging oxygen out of the medium, the reaction flask was attached to the condenser to reflux with the inert gas for 1 h at 80 °C. 5 mL of Te^−2^ precursor NaHTe was added into the solution and then the system was heated to 110C. Just after the temperature reached 110 °C, the growth of particles was observed by sampling. The solution started emitting green light under UV-irradiation after 10 minutes, because of the CdTe nano-colloids formation. The photostability was increased by coating CdTe nanocolloids with a proper, wider band-gap shell material. The band gap of CdTe is 1.5 eV. Cadmium sulfide (CdS), with a band gap of 2.5 eV, was chosen as the shell material for the cores, since, there is a small lattice parameter mismatch of 3.6% between CdTe and CdS. In ultra-pure water, adequate quantity of thiourea (CH_4_N_2_S) was dissolved and mixed with CdTe quantum dots. Te:S ratio was optimized to 1:10. Reflux was continued. Upon coating, red-shift in UV-visible and fluorescence spectra was observed simultaneously, because CdTe/CdS, when compared to CdTe, has a larger band gap energy and this observation indicated that core/shell structure formed instead of an alloy. Formation of CdTeS alloy would induce blue-shifting rather than red-shifting. Size of the quantum dots depends on the reflux time interval. Orange emitting core shell nanocolloids were obtained after 215 min reflux time.

**Figure 5:**
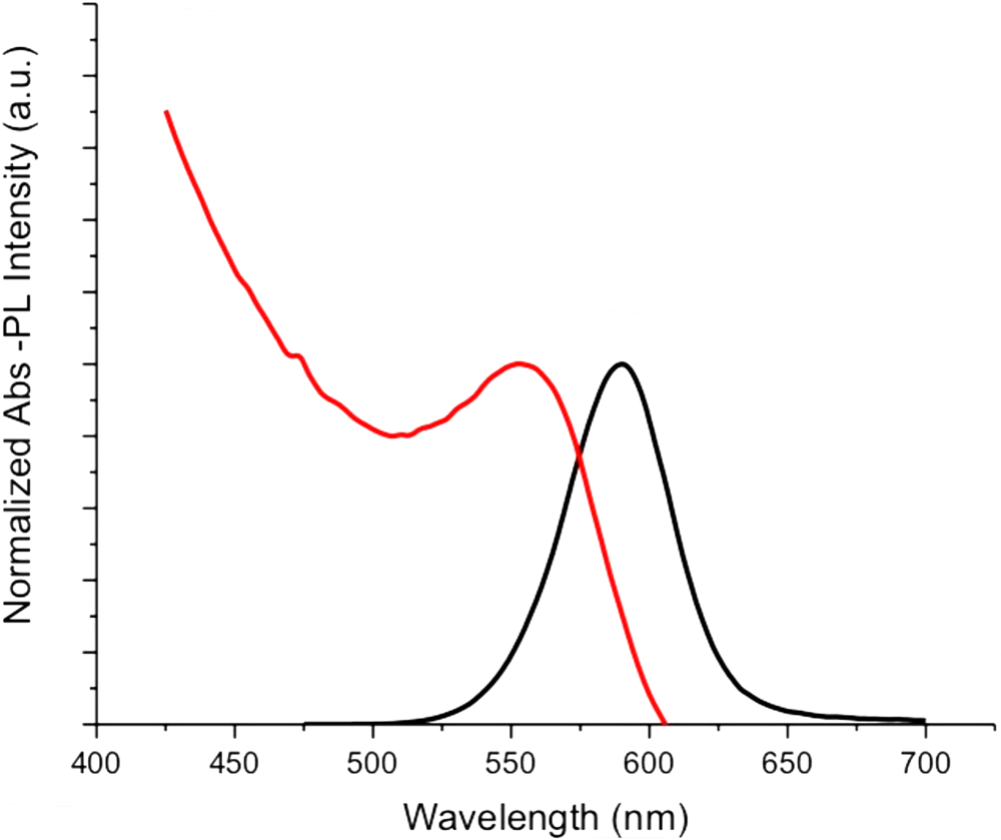
Normalized absorption and photoluminescence spectra of CdTe/CdS quantum dots.

The normalized absorption and photoluminescence (PL) spectra of the synthesized quantum dots were obtained as in Fig. 5 using Edinburgh Instruments FS5 Fluorospectrometer. Absorption spectrum exhibited the conventional quantum dot absorption behaviour of high absorption towards UV wavelengths and low absorption towards red wavelengths. The first excitonic peak was observed at 554 nm, therefore, the emission wavelength of the monochromator of the spectrofluorometer was set to 554 nm, and the fluorescence spectrum was measured as shown in Fig. 5. The emission was orange-colored. The spectrum with a full-width at half-maximum (FWHM) value of 45 nm, had a peak value at 590 nm. The Stokes shift of the synthesized quantum dots was 36 nm.

These quantum dots were chosen on purpose, since the wavelength of the laser source in TRFS experiments was suitable for the excitation of these dots.

### Acoustic Impedance Microscopy

**Figure 6:**
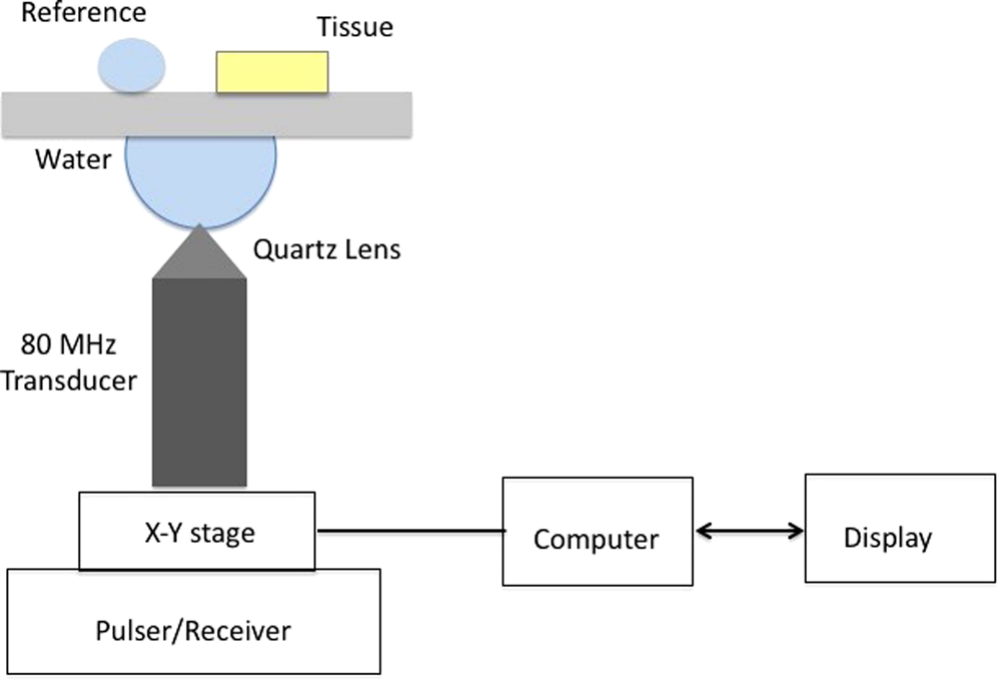
Schematic of SAM setup in acoustic impedance mode.

The methods were carried out in accordance with Bogazici University Institutional Review Board for Research with Human Subjects. The atherosclerotic plaques were characterized by scanning acoustic microscope (AMS-50SI) developed by Honda Electronics (Toyohashi, Japan). Figure 6 shows the schematic of SAM setup in acoustic impedance mode. It is mainly composed of a transducer with quartz lens, a pulser/receiver, an oscilloscope, a computer and a display monitor. 80 MHz transducer, which has a spot size of 17 *μ*m and a focal length of 1.5 mm, generates single pulses of width of 5 ns with a repetition rate of 10 kHz and also collects the reflected acoustic waves, therefore, acts as a pulser/receiver. Water is chosen to be the coupling medium between the quartz lens and the substrate. X-Y stage controlled by a computer is responsible in the two-dimensional scanning of the transducer. The reflected signals from both the reference and target material are analysed by the oscilloscope. Finally, acoustic intensity and impedance maps of the region of interest with 300 × 300 sampling points are visualized with a lateral resolution of approximately 20 *μ*m.

**Figure 7:**
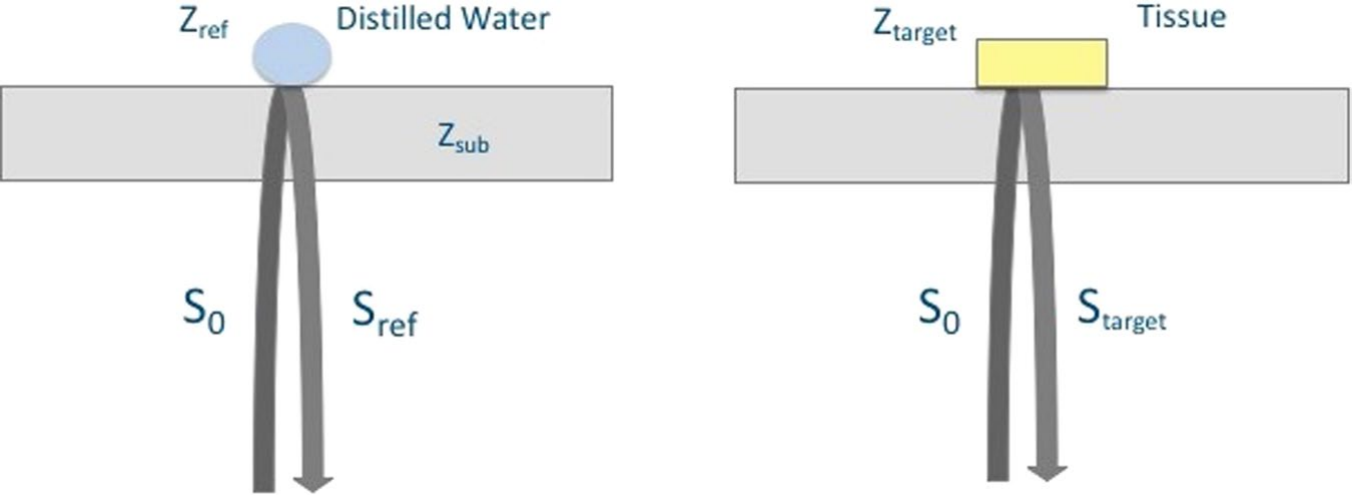
Principle of SAM in acoustic impedance mode. The acoustic waves reflected from the surfaces of distilled water and the tissue are collected by the same transducer and compared for the calculation of the acoustic impedance of the tissue.

Figure 7 shows the principle of SAM in acoustic impedance mode. The target is the tissue under investigation and as a reference, distilled water is widely used. The target signal is

1 $${S}_{target}=\frac{{Z}_{target}-{Z}_{sub}}{{Z}_{target}+{Z}_{sub}}{S}_{0}$$

where *S*_0_ is the signal generated by the 80 MHz transducer, *Z*_*target*_ is the acoustic impedance of the tissue and *Z*_*sub*_ is the acoustic impedance of the polystyrene substrate (2.37 MRayl). The tissue’s acoustic impedance is calculated by comparing the reflected signal from the tissue with the one from the reference. The reflected signal from the reference is

2 $${S}_{ref}=\frac{{Z}_{ref}-{Z}_{sub}}{{Z}_{ref}+{Z}_{sub}}{S}_{0}$$

where *S*_*ref*_ is the reference’s acoustic impedance (1.50 MRayl). Consequently, the target’s acoustic impedance can be written as

3 $${Z}_{target}=\frac{1+\frac{{S}_{target}}{{S}_{0}}}{1-\frac{{S}_{target}}{{S}_{0}}}{Z}_{sub}=\frac{1-\frac{{S}_{target}({Z}_{sub}-{Z}_{ref})}{{S}_{ref}({Z}_{sub}+{Z}_{ref})}}{1+\frac{{S}_{target}({Z}_{sub}-{Z}_{ref})}{{S}_{ref}({Z}_{sub}+{Z}_{ref})}}{Z}_{sub}$$

with a constant generated signal *S*_0_^22^.

### Alternative Sample Setup

**Figure 8:**
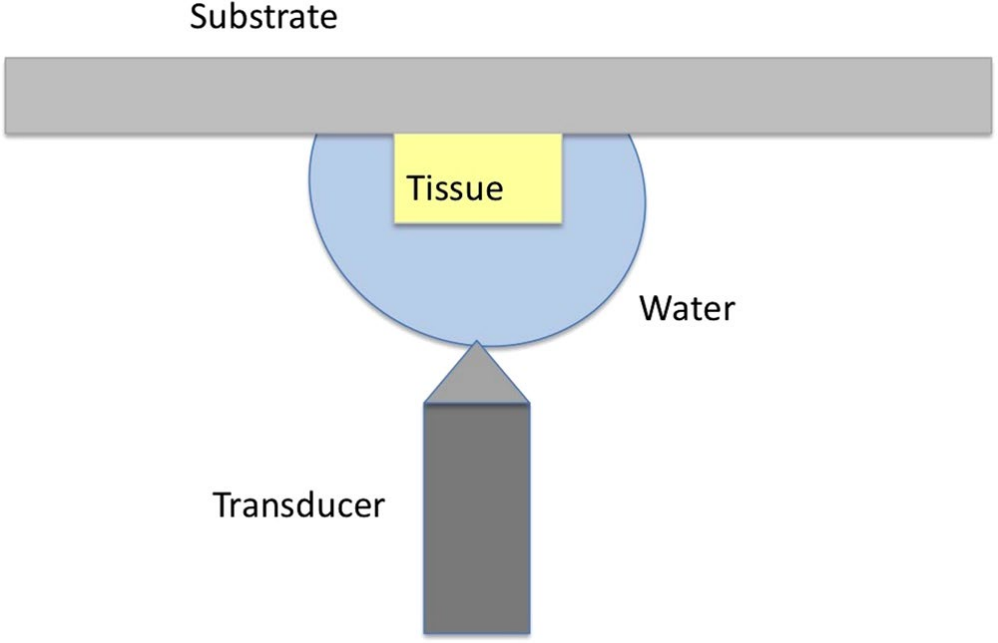
Alternative sample setup for the tissues.

The observation of soft tissues, which are installed into scanning acoustic microscope as shown in Fig. 6, is very simple, however, with harder tissues, such as tooth and bone, observation becomes difficult and sometimes impossible, since tissue surface and substrate surface do not cohere and air in between reduces the reflecting signal. For a solution, an alternative sample setup is adapted as shown in Fig. 8 for hard tissues^43^. The hard tissue attached to a polystyrene substrate is installed into scanning acoustic microscope such that, ultrasound signals generated by the transducer travel only through water and then are reflected back from the sample’s surface. The same equations for acoustic impedance calculation are valid, but water acts like the substrate and polystyrene substrate acts like the reference, instead.

### Statistical Analysis

Table 1 presents acoustic impedance microscopy results and Table 2 presents TRFS results in collagen-rich and calcific regions. Student’s t-test was used for the determination of statistical differences between these two regions and the level of statistical significance was set to p < 0.01.

### Time-resolved Fluorescence Spectroscopy

**Figure 9:**
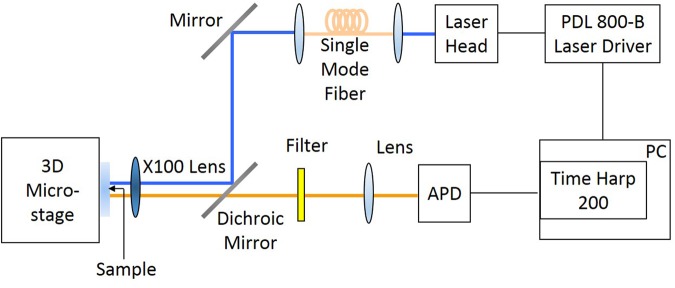
FLIM setup.

For time-resolved fluorescence spectroscopy measurements, a TimeHarp 200 PC-Board system (Picoquant, GmbH) is used. The optical setup used in our experiments is given in Fig. 9. The excitatiton source is a pulsed diode laser with a wavelength of 470 nm (LDH-D-C-470 Picoquant, GmbH). Gaussian beam illumination is obtained by launching the excitation beam into a single mode optical fiber (Thorlabs, S405-HP). For focusing the excitation light onto the samples and also collecting the emission of quantum dots on the samples, a microscope objective (Nikon ELWD 100x), whose numerical aperture is 0.70, is used. The excitation beam is eliminated completely by a long pass filter. A combination of long-pass and band-pass filter was interrogated into the optical system to allow detecting the fluorescence signals between wavelengths of 550 nm and 650 nm during TRFS measurements. Fluorescence lifetime of the quantum dots is determined using the FluoFit computer program for multi-exponential decay fitting.

The fluorescence intensity *I*(*t*) decay at time *t* can be written as

4 $$I(t)=\sum _{i=1}^{n}{A}_{i}exp(\frac{-t}{{\tau }_{i}})$$

where *τ*_*i*_ is ith component’s fluorescence lifetime and *A*_*i*_ is its amplitude. The fractional impact of the components to the total intensity can be written as

5 $${f}_{i}=\frac{{A}_{i}{\tau }_{i}}{\sum _{i}{A}_{i}{\tau }_{i}}$$

The intensity decay is defined as either the amplitude average lifetime or the intensity average lifetime. The amplitude average lifetime is defined as

6 $$ < \tau  > =\sum _{i}\,{f}_{i}{\tau }_{i}$$

and the intensity average lifetime is defined as

7 $$\bar{\tau }=\frac{{A}_{i}{\tau }_{i}}{\sum _{i}{A}_{i}}$$

## Data Availability

Corresponding author can provide the datasets of this study on reasonable request.
